# Effects of high-flow nasal cannula with oxygen on self-paced exercise performance in COPD: A randomized cross-over trial

**DOI:** 10.1097/MD.0000000000028032

**Published:** 2021-12-23

**Authors:** Ke-Yun Chao, Wei-Lun Liu, Yasser Nassef, Chi-Wei Tseng, Jong-Shyan Wang

**Affiliations:** a Department of Respiratory Therapy, Fu Jen Catholic University Hospital, Fu Jen Catholic University, New Taipei City, Taiwan; b School of Physical Therapy, Graduate Institute of Rehabilitation Sciences, Chang Gung University, Taoyuan, Taiwan; c Department of Emergency and Critical Care Medicine, Fu Jen Catholic University Hospital, Fu Jen Catholic University, New Taipei City, Taiwan; d School of Medicine, College of Medicine, Fu Jen Catholic University, New Taipei, Taiwan; e Institution of Medicine, Chung Shan Medical University, Taichung, Taiwan; f Heart Failure Center, Department of Physical Medicine and Rehabilitation, Chang Gung Memorial Hospital, Keelung, Taiwan; g Healthy Aging Research Center, Graduate Institute of Rehabilitation Science, Medical Collage, Chang Gung University, Tao-Yuan, Taiwan; h Research Center for Chinese Herbal Medicine, College of Human Ecology, Chang Gung University of Science and Technology, Tao-Yuan, Taiwan.

**Keywords:** 6-minute walking test, cardiopulmonary outcome, chronic obstructive pulmonary disease, heated humidified high-flow cannula

## Abstract

**Introduction:**

Studies have demonstrated that noninvasive ventilation improves exercise intolerance in patients with chronic obstructive pulmonary disease (COPD). The role of heated humidified high-flow nasal cannula (HFNC) therapy in patients with COPD on self-paced exercise performance remains unclear. Therefore, the purpose of the present study was to determine whether HFNC-aided supplemental oxygen during a 6-minute walk test (6MWT) would change self-paced exercise performance and cardiopulmonary outcomes in patients with stable COPD.

**Methods:**

A single-site, cross-over trial was conducted in a pulmonary rehabilitation outpatient department. This study enrolled 30 stable COPD patients without disability. The participants with and without HFNC performed 6MWTs on 2 consecutive days. Outcomes were the distance walked in the 6MWT, physiological, and cardiopulmonary parameters.

**Results:**

Those performing HFNC-aided walking exhibited a longer walking distance than those performing unaided walking. The mean difference in meters walked between the HFNC-aided and unaided walking scenarios was 27.3 ± 35.6 m (95% CI: 14.4–40.5 m). The energy expenditure index was significantly lower when walking was aided by HHHNFC rather than unaided (median: 1.21 beats/m walked vs median: 1.37 beats/m walked, *P* < .001). However, there were no differences in transcutaneous carbon dioxide tension between HHHNFC and non-HHHNFC patients.

**Conclusion:**

Walking distance and arterial oxygen saturation improved in stable COPD patients receiving HFNC with additional oxygen support. However, HFNC did not affect transcutaneous carbon dioxide tension and the self-reported dyspnea score during the walking test. The present study demonstrated the feasibility and safety of using HFNC in self-paced exercise.

**Trial registration:**

NCT03863821

## 1 Introduction

Chronic obstructive pulmonary disease (COPD) is characterized by persistent airflow limitation with chronic inflammation of the respiratory system.^[1,2]^ Patients with COPD often experience exercise limitation and physical inactivity due to muscular weakness and severe dyspnea. Symptoms of COPD may contribute to activity restriction, deconditioning, and exercise intolerance.^[3,4]^ O’Donnell et al have demonstrated that dynamic hyperinflation can cause dyspnea due to exertion in patients with COPD.^[5–7]^ Importantly, mismatching of energy demand and supply in ventilatory mechanics may lead to exercise-induced dyspnea.^[8]^ Growing evidence indicates that pulmonary rehabilitation improves the clinical outcomes, including walking distance in patients with symptomatic COPD.^[8–10]^ Furthermore, a 5-year observational study revealed that pulmonary rehabilitation coupled with negative pressure ventilation reduced the acute exacerbation rate, medical cost, and improved walking distance in patients with COPD.^[11]^

In 2002, the American Thoracic Society (ATS) published guidelines for the 6-minute walk test (6MWT), standardizing a step-by-step protocol and encouraging further application of the 6MWT. The 6MWT is a self-paced field test that measures the submaximal level of functional capacity and is an ideal examination for patients with chronic respiratory failure who are unable to reach their maximal exercise capacity.^[12]^ Walking is a submaximal level of exertion that most people can perform during daily activity. Therefore, functional capacity is more easily reflected by the 6MWT than by other examinations.^[12,13]^ The 6MWT is sensitive for evaluating disease severity and response to treatment for cardiovascular and respiratory diseases.^[14]^ Decreased distance in the 6MWT is associated with increased risks of morbidity and mortality in patients with pulmonary disorder.^[14–16]^ Cukier et al^[17]^ assessed the self-paced exercise capacity with 6WMT in patients with stable COPD. Noninvasive ventilation (NIV) has been demonstrated to improve exercise intolerance and health-related quality of life in patients with severe COPD.^[18,19]^ NIV can be used by patients with COPD and ventilatory dependence during walking to improve symptoms and increase the walking distance.^[20,21]^ However, the use of NIV as an adjunct to an exercise program is difficult and labor-intensive, especially in patients who have never experienced NIV or are intolerant to the interface and positive pressure.^[22,23]^ Studies have reported that the dropout rates from exercise programs with adjunct NIV ranged from 7.1% to 28%.^[24–26]^ Heated humidified high-flow nasal cannula (HFNC) delivers a flow rate of up to 60 L/min with adequate humidification and prescribed oxygen concentration.^[27–29]^ Studies have indicated that compared with NIV, HFNC enhances the tolerance because of increased comfort due to warm, and humidified gas inflow that facilitates bronchial hygiene.^[30–32]^ HFNC can be regarded as appropriate noninvasive respiratory support for patients with COPD during exercise. The use of HFNC has been reported to improve health status and exercise capacity.^[33,34]^ Suzuki et al^[35]^ showed HFNC was not superior to conventional oxygen in exercise capacity in patients with fibrotic interstitial lung disease. Cirio et al^[36]^ demonstrated that HFNC improves the self-reported dyspnea and fatigue score in patients with severe COPD and ventilatory limitation. Limited evidence is available regarding the application of HFNC in exercise training during pulmonary rehabilitation. Therefore, the purpose of the present study was to determine whether HFNC with additional supplemental oxygen on 6MWT would change the self-paced exercise performance and cardiopulmonary outcomes.

## 2 Methods

### 2.1 Participants

This investigation conducted a single-site, cross-over trial at the Pulmonary Rehabilitation Outpatient Department of Fu Jen Catholic University Hospital from August 2018 through November 2019. The study was approved by the institutional review board for human studies of Cathay General Hospital Taipei, Taiwan (CGHFJCUH107002), and was registered with ClinicalTrials.gov (NCT03863821). Informed written consent was obtained from all the participants.

Patients with confirmed diagnosis of COPD and those who underwent the pulmonary rehabilitation program for at least 1 month were eligible for enrollment. COPD was defined using the Global Initiative for Chronic Obstructive Lung Disease definition.^[37]^ Patients who had left-side heart failure, had COPD acute exacerbation within 3 months, received a diagnosis of neuromuscular disease, had an artificial airway, required mechanical ventilator or NIV support, or were unable to perform the 6MWT were excluded.

### 2.2 Intervention

**Figure 1:**
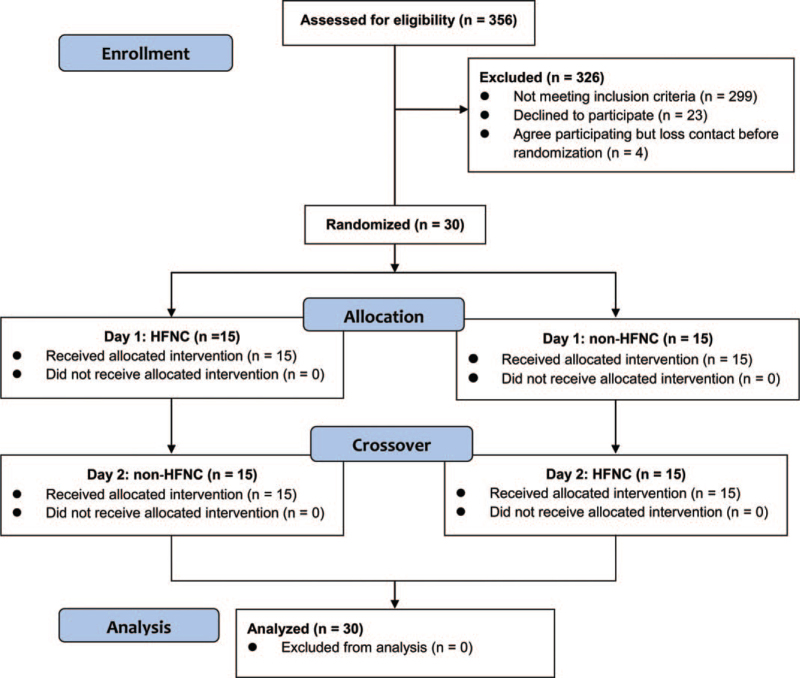
CORSORT diagram. HFNC = heated humidified high-flow nasal cannula.

Each participant performed 2 6MWTs on 2 consecutive days. On the first day, the participants were randomly assigned to either receive or not receive HHHNFC during the 6MWT. Each participant then repeated the test on the second day but under the opposite condition (Fig. 1). The randomization sequence was performed on the website http://randomization.com. In the HHHNFC-aided walking, the participants received supplemental oxygen (FiO_2_ = 0.4 and flow rate = 40 L/min) during the 6MWT. HFNC was delivered using the Airvo^2^ (Airvo^2^, Fisher & Paykel Healthcare, Auckland, New Zealand). The portable HFNC was powered by an external battery (TS1500C, OPTI-UPS, Taipei, Taiwan) during the 6MWT. The HFNC was provided through 3 sizes of nasal cannula (Optiflow, Fisher & Paykel Healthcare, Auckland, New Zealand). The outer diameter of the nasal cannula ranged from 40 to 70 mm. In the test in which HHHNFC was not received, the participants underwent the 6MWT alone. In accordance with the ATS protocol, an additional oxygen inflow (flow rate = 3 L/min) was delivered through a traditional nasal cannula if the oxygen saturation was < 88%. HFNC was removed immediately at the end of the 6MWT.

### 2.3 6MWT

The present study used a checklist for reporting the design of 6MWT in each COPD patient.^[38]^ The 6MWT was performed in accordance with the ATS guidelines.^[12]^ A straight, flat, 20-m-long corridor was used, which is shorter than that which is described in ATS guidelines, because of the site's limitations. Instructions were provided prior to the 6MWT and encouragement was provided during the 6MWT according to the ATS guidelines. All the 6MWTs were conducted by the same investigator. A trolley was used to carry the devices, including the Airvo^2^ with an external battery, oxygen cylinder, and monitors. The same research assistant was responsible for the trolley movement.

### 2.4 Outcomes measurement

The primary outcome was the 6-minute walk distance (6WMD) with or without HFNC. Heart rate (HR) and SpO_2_ were measured continually through a wrist-worn pulse oximeter (WristOx2, Nonin Medical, Plymouth, MN). All data were transferred through Bluetooth transmission, and variables were calculated using computer software (nVision Version 6.4, Nonin Medical, Plymouth, MN). A transcutaneous carbon dioxide tension (PtcCO_2_) monitor (TCM4, Radiometer, Medical AsP, Brønshøj, Denmark) that continually measured PtcCO_2_ by using an electrochemical transducer was employed. The measurement site on the participants was cleaned using an alcohol pad. Additionally, a noninvasive hemodynamics monitor that obtains measurements using electrical cardiometry was used (ICON, Osypka Medical, Berlin, Germany) before and immediately after the 6MWT. The participants were asked to rate their dyspnea on a 0 to 10 modified Borg scale, with 0 indicating “none” and 10 indicating “the worst.” Higher scores meant worse dyspnea. Physiological and respiratory parameters were collected 30 minutes before, during, and on completion of the 6MWT. To determine the energy cost of HFNC-aided walking and non-HFNC-aided walking, mean HR and walking speed were employed to calculate the energy expenditure index (EEI). In this research, higher EEI represents poor energy cost of walk.^[39]^

### 2.5 Statistical analysis

To determine the minimal sample size to ensure powerful testing of the intervention, 30 participants were required for the main analysis, as determined using a power of 0.8 with an α error of 0.05 according to previous findings.^[20]^ Subjects with COPD underwent the 6WMT with and without NIV (O_2_: 220 ± 84.8 m vs O_2_ + NIV: 260 ± 64.9 m). Statistical analyses were performed using IBM SPSS (version 22.0 for Windows, Chicago, IL). Because the sample size was small, the Wilcoxon signed-rank test was used for analyzing all variables. Statistical significance was indicated by *P* < .05. The results are presented as number (%), mean ± standard deviation, or median (interquartile range).

## 3 Results

Thirty participants without disability were enrolled in the present study (mean ± standard deviation age, 66.8 ± 8.4 years; forced expiratory volume in 1 s, 72.8 ± 22.2% [predicted]). Description of the demographic characteristics at baseline is provided in Table 1. All 30 participants completed the 6MWT on 2 consecutive days. During their unaided 6MWT, only 1 participant required additional oxygen support due to low SpO_2_. The mean difference in meters walked between the HFNC-aided and unaided walking scenarios was 27.3 ± 35.6 m (95%CI: 14.4–40.5 m; *P* < .001; Table 2).

**Table 1:** Characteristics of participants.

Subjects	30
Demographic data	
Gender, (male/female)	28/2
Age, yrs	67 (60.8–72)
BMI, kg/m^2^	24.2 (21.7–26.7)
Former smoker, (%)	28 (93.3)
Lung function	
FEV_1_, % predicted	78.5 (57.5–87.8)
FVC, % predicted	91.5 (79.5–104.3)
FEV_1_/FVC, %	63 (53–67)
RV, % predicted	142 (113–169)
TLC, % predicted	101 (89.3–105)
GOLD stage	
Stage I, (%)	16.0 (53.3)
Stage II, (%)	10.0 (33.3)
Stage III, (%)	4.0 (13.3)

Data are presented as median (IQR) or number (%). BMI = body mass index, FEV_1_ = forced expiratory volume in the first second, FVC = forced vital capacity, GOLD = Global Initiative for Chronic Obstructive Lung Disease, RV = residual volume, TLC = total lung capacity.

**Table 2:** Physiological parameters and walking distance before and after the 6MWT.

			Mean change (HFNC minus non-HFNC)		
	HFNC (n = 30)	Non-HFNC (n = 30)		95% CI	*P* value
6MWT outcome					
6WMD, m	454 (360–515)	430 (320–494)	27.3 ±35.6	14.1–40.5	< .001^‡^
Walking speed, m/min	75.7 (59.9–85.8)	71.6 (53.3–82.4)	4.54 ± 5.89	2.34–6.74	< .001^‡^
HR peak, b/m	109 (96–122.5)	112 (105.8–124.5)	−3.9 ± 12.6	−8.61 to 0.81	.072
SpO_2_ nadir, %	93 (90.8–94)	91.5 (87–94)	1.8 ± 3.65	0.47–3.2	.015^∗^
EEI, beats/meter walked	1.21 (1.13–1.61)	1.37 (1.21–1.57)	−0.13 ± 0.18	−0.19 to −0.06	< .001^‡^
Before 6MWT					
HR, b/m	78.7 (67.8–87.8)	77.2 (66.9–91)	1.12 ± 6.2	−1.20 to 3.43	.411
SpO_2_, %	94.9 (93.5–96.4)	95.0 (93.3–96.2)	0.19 ± 1.37	−0.33 to 0.7	.566
RR, b/m	17.5 (16.2–20)	17.5 (15.7–20)	0.21 ± 2.02	−0.55 to 0.96	.755
Borg-D	1 (0–2)	1.5 (0–2)		.658	
sBP, mm Hg	123 (118–138)	126 (117–139)	−4.35 ± 23.3	−13.1 to 4.36	.658
dBP, mm Hg	76 (70–84)	78 (69–86)	0.9 ± 7.08	−1.75 to 3.55	.416
MAP, mm Hg	92 (86–102)	95 (84–101)	−0.85 ± 9.68	−4.46 to 2.76	.805
PtcCO_2_, mmHg	40.5 (36.7–43.7)	39.7 (36.1–44)	0.55 ± 5.62	−1.55 to 2.65	.741
After 6MWT					
HR, b/m	93.5 (80–105)	92 (81.8–110)	0.07 ± 11.6	−4.28 to 4.41	.9
SpO_2_, %	97 (95–98)	95 (92.8–97)	2.3 ± 3.11	1.14–3.46	.001^†^
RR, b/m	23 (21–25.3)	25 (21.8–25)	−0.4 ± 3.3	−1.63 to 0.83	.574
Borg-D	5 (3–6.25)	5.5 (3–6)		.257	
sBP, mm Hg	138 (128–161)	145 (130–160)	−1.9 ± 18.4	−8.76 to 4.96	.354
dBP, mm Hg	82 (72–93)	83 (72–91)	0.28 ± 8.85	−3.03 to 3.58	.714
MAP, mm Hg	104 (91–115)	101 (93–113)	−0.45 ± 9.59	−4.03 to 3.13	.551
PtcCO_2_, mmHg	41.5 (36–46.3)	41.5 (36.8–45.3)	−0.16 ± 5.7	−2.29 to 1.97	.596

Data are presented as mean ± SD or median (IQR). 6MWT = six-minute walking test, 6WMD = six-minute walking distance, Borg-D = Borg dyspnea score, dBP = diastolic blood pressure, EEI = energy expenditure index, HFNC = high-flow nasal cannula, HR = heart beat, MAP = mean arterial pressure, PtcCO_2_ = transcutaneous carbon dioxide tension, RR = respiratory rate, sBP = systolic blood pressure, SpO_2_ = oxygen saturation.

∗ *P* < .05.

† *P* < .01.

‡ *P* < .001.

**Figure 2:**
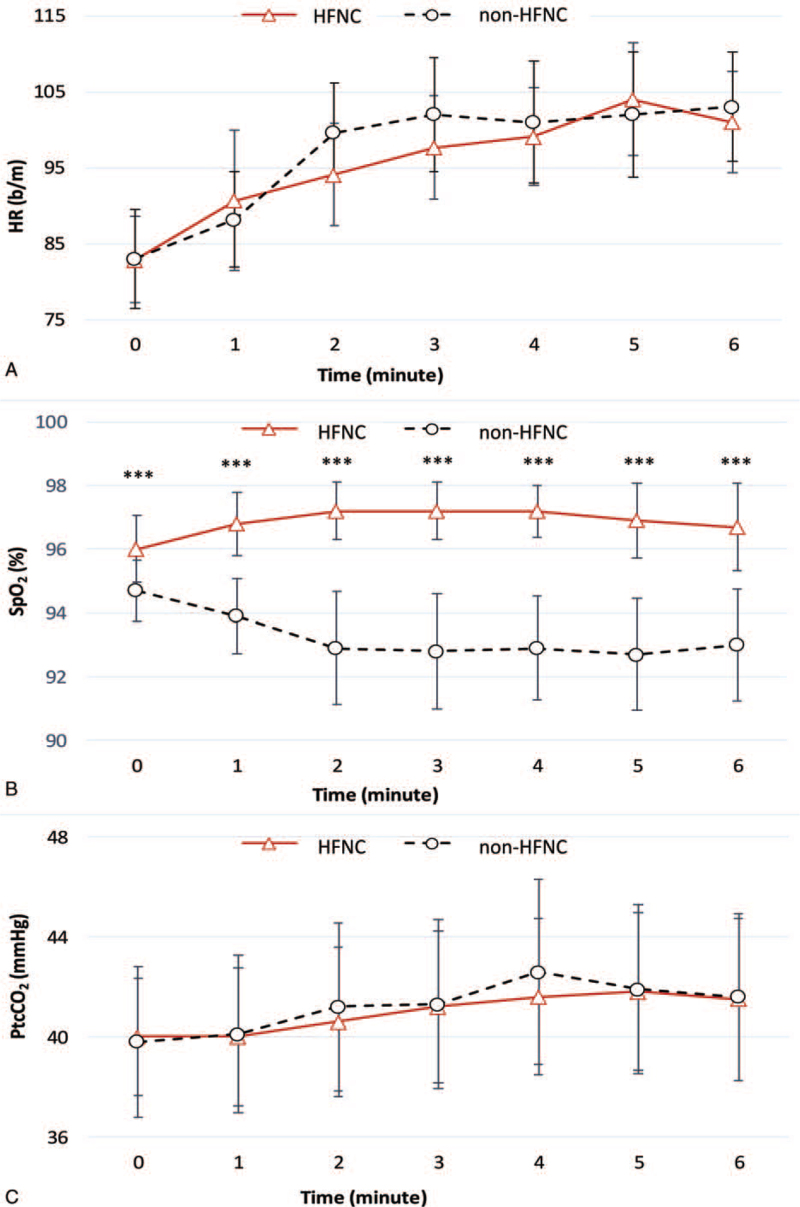
The physiological trend for (A) heart rate (HR), (B) oxygen saturation (SpO_2_), and (C) transcutaneous carbon dioxide tension (PtcCO_2_) during the 6MWT. ^∗∗∗^*P* < .001. 6MWT = six-minute walking test, HFNC = heated humidified high-flow nasal cannula.

The baseline parameters of the participants in the HHHNFC-aided and unaided -walking scenarios were comparable. The difference in SpO_2_ between the HFNC-aided and unaided walking scenarios was 2.3% ± 3.11% (95% CI: 1.14%–3.46%; *P* = .001). The EEI was significantly lower when walking was aided by HHHNFC rather than unaided (median: 1.21 beats/m walked [IQR: 1.13–1.61] beats/m walked vs median: 1.37 beats/m walked [IQR: 1.21–1.57] beats/m walked, *P* < .001). The differences in other parameters did not reach significance (Table 2). The difference in the peak HR during the 6MWT between the HHHNFC-aided and unaided walking scenarios was nonsignificant; however, the SpO_2_ nadir was significantly lower when walking was unaided (median: 91.5% [IQR: 87%–94%] vs median: 90.3% [IQR: 90.8%–94%], *P* = .015). A significant difference was observed between the SpO_2_ level during the 6MWT. By contrast, the changes in HR and PtcCO_2_ did not reach the significance level (Fig. 2 and Table 3). The hemodynamic parameters after the 6MWT performed under and not under HHHNFC were comparable (Table 4). No adverse events related to the present study were noted.

**Table 3:** Changes of HR, SpO_2_, and PtcCO_2_ during 6MWT.

			Mean change (HFNC minus non-HFNC)		
	HFNC (n = 30)	Non-HFNC (n = 30)		95% CI	*P* value
Change of HR					
HR at begging, b/min	80.5 (73.3–96)	82.5 (72–92.3)	0.03 ± 8.02	−2.96 to 3.03	.703
HR at 1-min, b/min	93.5 (79.3–102)	92 (79–99.3)	2.4 ± 22.7	−6.08 to 10.9	.734
HR at 2-min, b/min	97.5 (83.3–105)	102 (89–109)	−5.5 ± 15.5	−11.3 to 0.29	.045
HR at 3-min, b/min	98 (86.8–108)	103 (90.8–111)	−4.4 ± 13.5	−9.42 to 0.62	.07
HR at 4-min, b/min	97 (82.8–114)	101 (90.5–112)	−2.2 ± 16.1	−8.29 to 3.76	.432
HR at 5-min, b/min	103 (89.3–114)	105 (87–112)	1.7 ± 18.6	−5.25 to 8.65	.936
HR at 6-min, b/min	103 (87.5–112)	107 (92.5–112)	−1.9 ± 13.4	−6.88 to 3.14	.35
Change of SpO_2_					
SpO_2_ at begging, %	96 (94–98)	95 (93–96)	1.37 ± 1.54	0.79–1.94	< .001^∗^
SpO_2_ at 1-min, %	97 (95–98)	94 (91.8–96)	2.87 ± 2.18	2.05–3.68	<.001^∗^
SpO_2_ at 2-min, %	98 (96–98.3)	94 (89.8–96)	4.37 ± 3.25	3.15–5.58	< .001^∗^
SpO_2_ at 3-min, %	98 (96–98)	94 (89.8–96)	4.43 ± 3.39	3.17–5.7	< .001^∗^
SpO_2_ at 4-min, %	98 (96.8–98)	94 (90–95.3)	4.27 ± 3.35	3.01–5.52	< .001^∗^
SpO_2_ at 5-min, %	98 (96–98)	94 (89.8–96)	4.20 ± 3.52	2.89–5.51	< .001^∗^
SpO_2_ at 6-min, %	98 (95.8–98)	94 (90–96)	3.73 ± 3.82	2.31–5.16	< .001^∗^
Change of PtcCO_2_					
PtcCO_2_ at begging, mm Hg	40 (36.8–43)	40 (36–45.3)	0.27 ± 5.55	−1.80 to 2.34	.698
PtcCO_2_ at 1-min, mm Hg	41 (36.8–43.3)	40 (36.8–46)	0.1 ± 5.59	−1.99 to 2.19	.596
PtcCO_2_ at 2-min, mm Hg	42 (36–45)	41 (37.8–47.5)	−0.67 ± 6.44	−3.07 to 1.74	.321
PtcCO_2_ at 3-min, mm Hg	42.5 (36–46)	41 (37.8–47)	−0.13 ± 6.26	−2.47 to 2.2	.501
PtcCO_2_ at 4-min, mm Hg	45 (37–47)	42.5 (37.8–50.3)	−0.93 ± 7.65	−3.79 to 1.92	.366
PtcCO_2_ at 5-min, mm Hg	43.5 (37.8–47)	42 (37.8–47)	−0.17 ± 6.28	−2.51 to 2.18	.523
PtcCO_2_ at 6-min, mm Hg	42.5 (37–47.3)	42 (37–44.3)	−0.13 ± 6.20	−2.45 to 2.18	.555

Data are presented as mean ± SD or median (IQR). 6MWT = six-minute walking test, HFNC = high-flow nasal cannula, HR = Heart rate, PtcCO_2_ = transcutaneous carbon dioxide tension, SpO_2_ = oxygen saturation.

∗ *P* < .001.

**Table 4:** Hemodynamics outcomes.

			Mean change (HFNC minus non-HFNC)		
	HFNC (n = 30)	Non-HFNC (n = 30)		95% CI	*P* value
Before 6MWT					
SV, mL	48.7 (42.9–55.8)	49.6 (45.5–54.6)	0.86 ± 8.32	−2.25 to 3.97	.666
CO, L/min	3.94 (3.36 5.18)	3.99 (3.26–4.73)	0.2 ± 0.89	−0.13 to 0.53	.381
TFC, unit	16 (14.0–17.3)	16 (14.8–18)	−0.77 ± 3.04	−1.90 to 0.37	.22
ICON, unit	28.4 (22.5–39.9)	27.8 (23.8–37.2)	1.53 ± 10.7	−2.45 to 5.51	.399
FTC, ms	323 (316–331)	320 (316–329)	−1.53 ± 17.09	−7.91 to 4.85	.813
SVV, %	18 (13.8–21)	16.5 (13.0–22.5)	−0.4 ± 7.87	−3.34 to 2.54	.618
SVR, dynes · sec/cm^5^/m^2^	1744 (1334–2280)	1952 (1476–2430)	−95.2 ± 429	−256 to 65.1	.206
SVRI, unit	2775 (2331–4099)	3388 (2594–3985)	−145 ± 758	−429 to 138	.245
STR, unit	0.49 (0.44–0.53)	0.47 (0.41–0.5)	0.02 ± 0.08	−0.01 to 0.05	.265
PEP, ms	130 (119–138)	130 (117–138)	0.17 ± 17.3	−6.27 to 6.61	.861
LVET, ms	279 (252–301)	282 (259–304)	−3.47 ± 27.6	−13.8 to 6.85	.649
After 6MWT					
SV, mL	59.1 (49.9–68.7)	54.2 (52.3–62.4)	1.67 ± 12.3	−2.91 to 6.26	.622
CO, L/min	5.07 (3.86–6.86)	5.36 (4–6.39)	0.09 ± 1.98	−0.65 to 0.83	.886
TFC, unit	16 (14–18.3)	16 (14.8–18)	−0.27 ± 2.8	−1.31 to 0.78	.623
ICON, unit	44.2 (28.1–59.4)	39 (30–54)	−0.03 ± 23.2	−8.68 to 8.62	.758
FTC, ms	326 (314–330)	324 (312–330)	0.83 ± 17.01	−5.52 to 7.18	.967
SVV, %	22 (17.8–25.3)	20.5 (16–25.3)	0.1 ± 11.1	−4.04 to 4.24	.51
SVR, dynes · sec/cm^5^/m^2^	1487 (1046–1759)	1460 (1200–1777)	−36 ± 434	−198 to 126	.75
SVRI, unit	2596 (2014–2950)	2526 (2005–3169)	−80 ± 720	−349 to 189	.688
STR, unit	0.44 (0.41–0.52)	0.43 (0.36–0.5)	0.02 ± 0.08	−0.01 to 0.05	.235
PEP, ms	114 (100–129)	112 (102–131)	1.13 ± 16.4	−4.99 to 7.26	.75
LVET, ms	277 (240–291)	260 (233–291)	6.23 ± 28.7	−4.49 to 17	.233

Data are presented as mean ± SD or median (IQR). 6MWT = six-minute walking test, CO = cardiac output, FTC = correct flow time, HFNC = high-flow nasal cannula, ICON = index of contractility, LVET = left ventricular ejection time, PEP = preejection period, STR = systolic time ratio, SV = stroke volume, SVV = stroke volume variation, TFC = thoracic fluid content.

## 4 Discussion

In patients with stable COPD, the use of HFNC with additional oxygen support during the walking test resulted in significantly increased walking distance. Reports on the 6MWT indicated that the minimal clinically important change in distance was > 30 m in patient response to pulmonary rehabilitation^[40]^ and ranged from 14 to 30.5 m across multiple patient groups.^[41]^ The difference of 6WMD caused by HFNC was 27.3 m (95% CI: 14.1–40.5 m) in the present study. HFNC patients had significantly higher arterial oxygen saturation with additional oxygen support during the 6MWT than non-HFNC patients. Additionally, EEI is used to evaluate the economy of walking at different speeds based on heart rate and oxygen intake. In this study, HFNC lowered EEI in patients with stable COPD.

On the other hand, no significant difference in PtcCO_2_ and breathing frequency was observed between HFNC and non-HFNC patients in this investigation, which may have indicated rapid washout of CO_2_. PtcCO_2_ was suggested in 1 report to be highly heterogeneous among patients with severe COPD during the 6WMT.^[42]^ Mauri et al demonstrated that HFNC enhanced CO_2_ clearance by reducing the respiratory rate and minute ventilation at similar arterial CO_2_ tension and pH level compared with conventional oxygen therapy in critically ill hypoxemic patients.^[43,44]^

Menadue et al^[45]^ reported that NIV during exercise training improves the percentage change in the peak, endurance exercise capacity and improves the physiological training effect. NIV during lower limb exercise training may help patients with COPD to exercise at a higher training intensity.^[45]^ However, to maintain the treatment pressure level of NIV, the mask must be tight-fitting, which may lead to intolerance of NIV. Two studies reported a 25% to 28% dropout rate due to NIV intolerance.^[24,46]^ HFNC causes less discomfort and irritation than NIV,^[30,31]^ improving adherence during exercise training. Similar to the observations of Cirio et al,^[36]^ no participants dropped out due to intolerance of HFNC in the present study. The 6MWT is a self-paced examination through which it is difficult to assess the outcomes of higher strength level exercises. Therefore, no significant change was observed in the hemodynamic parameter. Dreher et al^[20]^ used NIV during the walking test and asked participants to self-move the rollator. A report indicated that use of a rollator improves walking distance even without ventilatory support in patients with COPD.^[47]^ By contrast, the 6WMD was decreased by 14% to 22% when patients with severe respiratory disability carried an air container.^[48]^ The trolley used in this work was moved by the research assistant, thus diminishing its influence on results.

The present study had limitations in several aspects. First, the use of additional oxygen support may have affected the outcomes. Oxygen is an independent variable affecting the 6MWT results of patients with COPD or interstitial lung disease.^[48–50]^ The 6MWD may have been increased by acute administration of oxygen.^[14]^ In the present study, none of the participants had received long-term oxygen therapy in their daily life. Second, we recruited patients with mild to moderate COPD; the results of the present study cannot be directly transferred to patients with severe COPD. Third, the 6MWT only reflects functional capacity; it cannot measure exercise capacity.^[51]^ Exercise capacity tests were conducted using a cycle ergometer^[52,53]^ or treadmill.^[54,55]^ Fourth, HFNC is an open system, and it is difficult to simultaneously use pneumotachographs and plethysmography due to the device's limitations. Neither minute volume nor respiratory system pressure was measured in the present study. Therefore, information regarding CO_2_ clearance was not obtained. Transcutaneous O_2_ and CO_2_ monitoring presented a decent signal quality index, whereas noninvasive cardiometry was unable to obtain data during the exercise movement. The sensor lines of the monitors and HFNC circuit may have caused interference for participants during the walking test. The wrist-worn pulse oximeter was the only device with Bluetooth transmission in the present study. Fifth, the present study demonstrated the short-term effect of HFNC on cardiopulmonary exercise performance in patients with COPD. However, the long-term effect remains unclear.

## 5 Conclusion

Application of HFNC with additional oxygen support improved the self-paced exercise performance by increasing walking distance and arterial oxygen saturation with unaltered PtcCO_2_ during the 6MWT in COPD patients. A lower energy cost was also observed in those performing HFNC-aided walking. Therefore, these findings suggest that the use of HFNC makes it feasible and safe to perform physical activity in patients with COPD. However, the application of HFNC in pulmonary rehabilitation warrants further research with long-term follow-up to determine the effects of regular exercise training with HFNC.

## Acknowledgments

This manuscript was edited by Wallace Academic Editing. This work was supported by a research grant from Fu Jen Catholic University Hospital (PL-201808008-M). This funding source had no role in the design of this study and did not have any role during its execution, analyses, interpretation of the data, or decision to submit results.

## Author contributions

**Conceptualization:** Ke-Yun Chao, Wei-Lun Liu, Jong-Shyan Wang.

**Data curation:** Ke-Yun Chao, Chi-Wei Tseng.

**Formal analysis:** Ke-Yun Chao, Yasser Nassef, Chi-Wei Tseng.

**Funding acquisition:** Ke-Yun Chao.

**Investigation:** Ke-Yun Chao, Wei-Lun Liu.

**Methodology:** Ke-Yun Chao, Wei-Lun Liu, Yasser Nassef, Jong-Shyan Wang.

**Project administration:** Ke-Yun Chao, Chi-Wei Tseng.

**Supervision:** Wei-Lun Liu, Jong-Shyan Wang.

**Validation:** Ke-Yun Chao, Wei-Lun Liu, Yasser Nassef, Jong-Shyan Wang.

**Writing – original draft:** Ke-Yun Chao.

**Writing – review & editing:** Ke-Yun Chao, Yasser Nassef, Jong-Shyan Wang.
